# Risk Factors for Recurrent Tracheoesophageal Fistula After Gross Type C Esophageal Atresia Repair

**DOI:** 10.3389/fped.2021.645511

**Published:** 2021-05-13

**Authors:** Shen Yang, Siqi Li, Zhi Yang, Junmin Liao, Kaiyun Hua, Yanan Zhang, Yong Zhao, Yichao Gu, Shuangshuang Li, Jinshi Huang

**Affiliations:** ^1^National Center for Children's Health, Department of Neonatal Surgery, Beijing Children's Hospital, Capital Medical University, Beijing, China; ^2^Department of Neonatal Surgery, The Affiliated Children's Hospital of Nanchang University, Nanchang, China

**Keywords:** recurrent tracheoesophageal fistula, esophageal atresia, risk factors, surgery, complication

## Abstract

**Background:** To determine the possible risk factors of recurrent tracheoesophageal fistula (rTEF) after Gross type C esophageal atresia (EA) and tracheoesophageal fistula (TEF) repair.

**Methods:** The medical records of 343 pediatric patients with Gross type C EA/TEF who underwent surgical repair were retrospectively analyzed. The patients were retrospectively divided into two groups according to whether they had rTEF. Univariate and multivariable logistic regression analysis were performed to identify risk factors for rTEF.

**Results:** After the diagnosis of EA/TEF, 343 patients (221 boys) underwent primary repairs after birth. According to the follow-up results (257 patients survived, 42 died, and 43 were lost to follow-up), 259 patients (257 survived and two died after rTEF repair) were included in the analysis. rTEF occurred in 33 patients (33/259, 12.74%), with a median onset time to recurrence of 3.8 (2.2, 8.2) months. Multivariate analysis showed that closing the original TEF with ligation and hospital stay ≥ 28.5 days were significant risk factors of rTEF with OR of 4.083 (1.481, 11.261) and 3.228 (1.431, 7.282).

**Conclusions:** Surgical closure technique of original TEF and the length of initial stay could influence the occurrence of rTEF after Gross type C EA/TEF repair.

## Introduction

Esophageal atresia (EA) and tracheoesophageal fistula (TEF) is one of the most common congenital malformations of the esophagus, with an incidence of 1/2500 – 1/4500 (1). The survival rate of Gross type C EA/TEF without severe malformation reported in the relevant literature is higher than 90% (1). Although survival rate has significantly improved, the procedure still has many postoperative complications. Recurrent tracheoesophageal fistula (rTEF) occurs in 3–14% of EA/TEF repairs (2). rTEF is difficult to diagnose and treat; complex reoperative surgery is usually required, but is associated with a high rate of secondary recurrence, morbidity, and mortality (3). Understanding the risk factors for rTEF is crucial to prevent its occurrence. In this study, we retrospectively reviewed patients with Gross type C EA/TEF from two tertiary children's hospital in China to determine the possible risk factors of rTEF.

## Materials and Methods

### Patients

The medical records of 343 pediatric patients with Gross type C EA/TEF who underwent surgical repair from January 2007 to January 2020 at Beijing Children's Hospital (*n* = 195) and from January 2013 to January 2020 at The Affiliated Children's Hospital of Nanchang University (*n* = 148) were retrospectively analyzed. The primary operations were performed *via* thoracoscopic or open approach. Demographic information, preoperative assessments, surgical notes, and information pertaining to surgical complications were extracted from the electronic medical records and analyzed. The symptoms that led to diagnosis of rTEF were recurrent pneumonia, coughing during feeding, or both. Diagnostic investigations for rTEF included bronchoscopy and esophagram. Bronchoscopy was used to measure the distance between the fistula and the tracheal carina and the glottis. The patients were retrospectively divided into two groups according to whether they had rTEF. Patients with missed diagnosis of the proximal TEF during the initial operation were excluded in this study. All patients in the rTEF group received surgical repair. This retrospective study was approved by the Medical Ethics Committee of the Beijing Children's Hospital (2019-k-333), and the patient informed consent requirements were waived.

### Statistical Analysis

Statistical analysis was performed by SPSS 23.0. Continuous variables were presented as the mean with standard deviation or median and interquartile range if the normality hypothesis test rejected the null hypothesis of normal distribution. Categorical variables were reported as counts and percentages. Pearson's χ^2^ test, Fisher's exact test, two independent samples *t*-tests and the non-parametric Mann-Whitney *U*-test were used to compare characteristics between the rTEF and non-rTEF groups. Univariate and multivariable logistic regression analysis were performed to identify risk factors for rTEF. *P* < 0.05 was considered statistically significant.

## Results

### Patient Characteristics

In this study, 343 patients were included (221 boys and 122 girls). These patients had a median gestational age of 38.1 weeks (range: 30–44 weeks) and a median birth weight of 2,850 g (range: 1,500–4,500 g). Two hundred and seventy-four patients were found to have congenital diseases, including non-syndromic anomalies (*n* = 252), VACTERL syndrome (*n* = 19), chromosome abnormality (*n* = 2), and other syndromic diagnosis (*n* = 1).

After the diagnosis of EA/TEF, all patients underwent primary repairs after birth. The primary operations were performed *via* thoracoscopic (*n* = 214, including eight that converted to an open thoracotomy) or open (*n* = 129) approach.

After a median follow-up of 16 months (range: 0 months to 163 months), 257 patients survived, 42 died and 44 patients were lost to follow-up. Among the 42 who died, parents of 21 patients had refused further treatment after surgical repair of EA/TEF. Thirteen patients died in hospital after surgical repair of EA/TEF, two died after rTEF surgery, one died of perforation after dilation procedure, and one died of cardiac abnormalities. The reason for death in the remaining four patients was unknown.

### Comparison of Clinical Characteristics Between the rTEF and Non-rTEF Groups

According to the follow-up results, 259 patients (257 survived and two died after rTEF repair) were included in the analysis of risk factors of rTEF. rTEF occurred in 33 patients (33/259, 12.74%), with a median onset time to recurrence of 3.8 (2.2, 8.2) months. Table 1 shows the comparison of clinical characteristics between the rTEF and non-rTEF groups. The method for original fistula closure (*P* = 0.044) and the length of hospital stay (*P* = 0.009) was associated with rTEF after the primary repair of Gross type C EA/TEF. There were no differences between the two groups for other clinical characteristics (all *P* > 0.05).

**Table 1:** Comparison between rTEF and non-rTEF groups.

**Variables**		**rTEF (*n* = 33)**	**Non-rTEF (*n* = 226)**	**Results**	***p***
Sex (*n*, %)	Male	24 (72.7)	143 (63.3)	1.123	0.289
	Female	9 (27.3)	83 (36.7)		
Age at first surgery (median, days)		5 (4, 8)	4 (3, 7)	−1.294	0.196
Gestational age (*n*, %)	Preterm	2 (8.0)	32 (19.3)	2.200	0.316
	Term	23 (92.0)	128 (77.1)		
	Overdue	0	6 (3.6)		
Birth weight (median, kg)		2.9 (2.5, 3.2)	2.9 (2.5, 3.2)	0.521	0.471
Distance (median, cm)		1.9 (1.1, 2.5)	1.8 (1.0, 2.5)	−0.604	0.546
Associated anomalies (*n*, %)	Non-syndromic	31 (93.9)	217 (96.0)	1.493	0.670
	VACTERL syndrome	2 (6.1)	8 (3.5)		
	Chromosome abnormality	0	1 (0.4)		
Type of surgery (*n*, %)^*^	Thoracoscopy	27 (81.8)	146 (64.6)	3.848	0.050
	Open surgery	6 (18.2)	80 (35.4)		
Method for fistula closure (*n*, %)	Transfixing suture	6 (18.8)	87 (39.2)	6.253	0.044
	Ham-lock clip	9 (28.1)	61 (27.5)		
	Ligation	17 (53.1)	74 (33.3)		
Operative time (median, min)		125 (100, 203)	126 (110, 180)	−0.257	0.797
Days until starting liquid diet (median, day)		10 (8, 15)	11 (8, 15)	−0.306	0.759
Duration of mechanical ventilation (median, day)		96 (39, 151)	86 (13, 141)	−1.005	0.315
Duration of intensive care unit (median, day)		7 (5, 13)	9 (6, 16)	−1.052	0.293
Hospital stay (median, day)		24 (20, 39)	21 (17, 27)	−2.616	0.009
Pneumothorax (*n*, %)	Yes	16 (48.5)	88 (38.9)	1.092	0.296
	No	17 (51.5)	138 (61.1)		
Anastomotic leakage (*n*, %)	Yes	8 (24.2)	41 (18.1)	0.699	0.403
	No	25 (75.8)	185 (81.9)		
Anastomotic stricture (*n*, %)	Yes	22 (68.8)	128 (63.7)	0.309	0.578
	No	10 (31.3)	73 (36.3)		
The number of dilations (median, *n*)		3 (2, 5)	5 (2, 9)	−1.915	0.056

*P value significant at <0.05. rTEF, recurrent tracheoesophageal fistula*.

* *Conversion to open surgery was included in the group of thoracoscopy*.

### Risk Factors of rTEF

In order to find the risk factors for rTEF, we conducted a multivariate analysis. As shown in Table 2, closing the fistula with ligation and hospital stay ≥ 28.5 days were significant risk factors of rTEF with OR of 4.083 (1.481, 11.261) and 3.228 (1.431, 7.282), respectively.

**Table 2:** Multivariate logistic regression analysis of prediction of rTEF.

**Variables**	**Estimate**	**Standard error**	**Wald**	***P***	**OR**
Closing TEF with Ham-lock clip	0.985	0.570	2.989	0.084	2.677 (0.877, 8.175)
Closing TEF with ligation	1.407	0.518	7.389	0.007	4.083 (1.481, 11.261)
Hospital stay ≥ 28.5 days^*^	1.172	0.415	7.975	0.005	3.228 (1.431, 7.282)

*rTEF, recurrent tracheoesophageal fistula*.

* *Stratification values for hospital stay was calculated by ROC curve analyses*.

## Discussion

rTEF is a serious complication after EA/TEF repair, with an incidence of 3–14% (2). Complex reoperation is usually required to address rTEF, and is associated with a high rate of complications. It is important to understand the influencing factors of rTEF in order to prevent its occurrence. However, literatures on factors affecting rTEF are rare. In this study, we retrospectively reviewed the cohorts of patients with Gross type C EA/TEF from two tertiary children's hospital in China and found that surgical closure technique of original TEF and the length of initial stay could influence the occurrence of rTEF after Gross type C EA/TEF repair.

Patients with rTEF usually present with choking and recurrent pneumonia. However, these symptoms may also appear due to other complications after the operation of EA/TEF, such as esophageal stricture or gastroesophageal reflux. It is difficult to diagnose rTEF by symptoms alone and it is usually necessary to rely on auxiliary examinations such as bronchoscopy, esophagography, and esophageal gastroscopy to form a diagnosis (4, 5). Repairing rTEF through thoracotomy or thoracoscopic surgery is the most reliable treatment, but it can also be treated using endoscopic therapies, tissue adhesives or de-epithelializing agents (4, 5). Conservative treatment is generally required, and surgery is performed when the child's lung infection and systemic nutritional status permits (3, 6). A systematic review reported that the probability of a second recurrence after thoracotomy repaired rTEF was 21%, the incidence of postoperative leakage was 16%, and the postoperative mortality rate was 3.7% (6). Therefore, understanding the risk factors of rTEF is of great significance to prevent the occurrence of rTEF.

Few studies have reported the influencing factors of rTEF. According to previous reports, rTEF may be associated with premature delivery, low birth weight (7), anastomotic leakage, anastomotic stricture (8, 9) and continuous esophageal dilation (2). Vered et al. reported that patients with rTEF had significantly more hospitalizations for respiratory symptoms and significantly more episodes of clinical bronchiolitis. In addition, the patients with rTEF had markedly more episodes of positive polymerase chain reaction for viruses (10). In this study, we found that rTEF was associated with the method for original fistula closure and the length of hospital stay. Other clinical features, perioperative conditions, and postoperative complications of EA/TEF were not significantly correlated with the occurrence of rTEF.

There is no unanimity on fistula closure technique for the primary repair of EA/TEF. In our research, the method of fistula closure has evolved from ligation to Ham-lock clip and then to transfixing suture. During thoracotomy, we use ligation (*n* = 4) or sutures (*n* = 80) to close the fistula. Ligation is used to ligate, and suture is used to transfix the tracheal end of the fistula, both of which occur before eventually cutting the fistula off. For thoracoscopic surgery, we use ligation (*n* = 86), ham-lock clips (*n* = 70), or sutures (*n* = 10) to close the fistula. The use of ligation and sutures is the same as during thoracotomy. The Ham-lock clip is used to clip and close the fistula near the trachea before cutting it off. We found that separating the fistula after ligation was an important risk factor for recurrence. In early EA/TEF repair, the fistula was simply ligated and an esophagus end-to-side anastomosis was performed, but it was believed that the fistula may be recanalized after surgery (11). Subsequently, surgeons tended to double-ligate and divide the fistula instead, followed by an end-to-end esophagus anastomosis. The incidence of rTEF after surgery ranges from 3 to 22% (12). Closing the fistula with transfixing suture can effectively reduce the incidence of rTEF. The European Reference Network for Rare Inherited Congenital Anomalies (ERNICA) Consensus Conference on the management of patients with EA/TEF recommends the use of suture to close the fistula (13). Previous research believed that there is an obvious risk of migration of the clip through the wall of the fistula and the development of a recurrent fistula (14), but our results show that using Ham-lock clip to close the fistula is not a risk factor for rTEF. Schlesinger et al. (15) reported usage of surgical clips to close the fistula in 67 patients, and only two patients subsequently developed rTEF. Nonetheless, the safety and effectiveness of surgical clips need further research before widespread clinical application.

We found that the length of hospital stay during the primary repair of EA/TEF was significantly longer in patients with rTEF than that of patients without rTEF. Although postoperative complications such as anastomotic leakage, pneumothorax, and anastomotic stricture were not significant risk factors of rTEF in this study, abscess formation, pneumonia, and the above complications may prolong hospital stay. Furthermore, premature is also associated with longer hospital stays (Supplementary Table 1). Therefore, prolonged hospital stay is a comprehensive reflection of postoperative complications and the recovery process. Due to the limitations of retrospective study case records, this study did not include abscess formation and pneumonia as factors; further research should be carried out for better understanding.

Due to the high proportion of recurrence of TEF after rTEF repair, the intraoperative skills and perioperative management of rTEF repair are very important. The technique currently adopted by our hospital is to use an absorbable 5-0 monofilament thread to sew up and close the tracheal end of the fistula before cutting the fistula off. Both incised ends of the fistula are then respectively closed using three interrupted 5-0 sutures (the tracheal end of the fistula is sutured twice to ensure that it is closed completely and to prevent air from escaping). Finally, the free part of the prevertebral fascia is placed between the two ends of the fistula to isolate the ends, which is known to prevent re-recurrence of the fistula. At present, it is generally believed that it is very important to choose a suitable tissue liner between the incised TEF during the rTEF operation to improve the success rate and avoid recurrence. Whether the application of this technique can reduce the occurrence of rTEF is still worthy of future research.

Basing on this is a retrospective study, surgical procedures and details, and perioperative management will change over time, and access to surgical details and postoperative complications information is also limited. Surgery performed by different surgeons and the respective follow-up time is different, resulting in a certain degree of heterogeneity in the results. Due to the small sample size of the two centers, the conclusions cannot be verified externally. We need to prospectively recruit more patients for regular and longer follow-up and obtain more detailed surgery and perioperative records to further understand the long-term prognosis of these patients and analyze risk factors for rTEF.

In conclusion, using transfixing suture to close TEF is an important protective factor for rTEF. We should avoid simply ligating the fistula during primary repair of EA/TEF. Furthermore, patients with long postoperative hospital stay (≥ 28.5 days) and postoperative complications should be highlighted for the possibility of rTEF during follow-up.

## Data Availability Statement

The original contributions presented in the study are included in the article/Supplementary Material, further inquiries can be directed to the corresponding author/s.

## Ethics Statement

The studies involving human participants were reviewed and approved by the Medical Ethics Committee of the Beijing Children's Hospital. Written informed consent from the participants' legal guardian/next of kin was not required to participate in this study in accordance with the national legislation and the institutional requirements.

## Author Contributions

JH, SY, and SiL: study conception and design. ZY, JL, KH, and YG: data acquisition. YZhan, YZhao, and ShL: analysis and data interpretation. SY and SiL: drafting of the manuscript. All authors contributed to the article and approved the submitted version.

## Conflict of Interest

The authors declare that the research was conducted in the absence of any commercial or financial relationships that could be construed as a potential conflict of interest.
