# Supplementary material for: Risk Factors for Recurrent Tracheoesophageal Fistula After Gross Type C Esophageal Atresia Repair
Source: Front Pediatr. 2021 May 13;9:645511. doi: 10.3389/fped.2021.645511 (PMC8155366; doi:10.3389/fped.2021.645511)
Supplement: Supplementary file 1 [file Table_1.DOCX]

Supplementary Table 1. Clinical comparison between premature and non-premature infants.

| Variables | | Premature infants (n =34) | Non-premature infants (n = 157) | Results | *p* |
| --- | --- | --- | --- | --- | --- |
| Hospital stay (median, day) |  | 24 (18, 31) | 20 (17, 26) | -1.965 | 0.049 |
| Associated anomalies (n, %) | Yes | 30 (88.2) | 128 (81.5) | 0.473 | 0.492 |
|  | No | 4 (11.8) | 29 (18.5) |  |  |
